# Synthesising evidence for equity impacts of population-based physical activity interventions: a pilot study

**DOI:** 10.1186/1479-5868-10-76

**Published:** 2013-06-15

**Authors:** David K Humphreys, David Ogilvie

**Affiliations:** 1 UKCRC Centre for Diet and Activity Research (CEDAR), Institute of Public Health, University of Cambridge, Box 296, Forvie Site, Cambridge, USA; 2 MRC Epidemiology Unit, Institute of Public Health, Cambridge, USA

**Keywords:** Physical activity, Inequalities, Interventions, Research synthesis

## Abstract

**Background:**

This study applied an equity lens to existing research to investigate what is known about the impact of population-level physical activity interventions on social inequalities.

**Methods:**

We performed a pilot systematic review to assess the availability of information on the social distribution of intervention effects, the targeting or allocation of interventions, and the baseline characteristics of participants. This comprised (i) a rapid review of systematic reviews and (ii) a review and synthesis of a sample of primary studies included in the eligible systematic reviews.

**Results:**

We found 19 systematic reviews of environmental and policy interventions. Relatively few of these (26%, n=5) were prospectively designed to examine effects on inequalities, and none were able to fully synthesise evidence of distributional effects. Over 40% of primary studies reported subgroup intervention effects; 18% reported socio-demographic interaction effects. Studies most often compared effectiveness by gender, followed by age, ethnicity, and socio-economic status. For gender, effects appeared to be evenly distributed overall, although heterogeneity in gradients between studies suggested that some interventions affect males and females differently.

**Conclusions:**

Our findings suggest that it is feasible to generate better evidence about how public health interventions may affect health inequalities using existing data and innovative methods of research synthesis.

## Background

Physical inactivity is a growing public health concern for societies and governments across the world [1]. Inactive lifestyles contribute to the aetiology of a number of chronic diseases including cardiovascular disease, type 2 diabetes, stroke and several cancers [2]. Participation in 30 minutes of moderate to vigorous physical activity on most days of the week may be sufficient to achieve health benefits [3]. However, findings from global and national health surveys reveal large proportions of the population failing to meet recommended levels of physical activity [4]. Furthermore, there is growing evidence to suggest that the prevalence of physical inactivity may be greater within some disadvantaged social groups [5].

Inequalities in the determinants of health, such as physical activity, are one of the main challenges for public policy [6]. Health inequalities are differences in health between and within populations [7]. Some health inequalities, such as the difference in life expectancy between males and females, may be largely attributable to biological characteristics [8]. However, inequalities can also result from characteristics of the environment that determine the health status of some population groups [9]. For example, inner city populations may have less access to safe environments that support the maintenance of active lifestyles [10]. Social inequalities are considered to be unfair, unjust, and avoidable [11], and consequently an important target for improvement.

Evidence from the World Health Organisation (WHO) observatory shows a clear social gradient in levels of physical inactivity when stratified by national income levels and gender [12]. Likewise, national surveys in England, Scotland and the United States show differences in physical activity between social groups defined in terms of gender, age, ethnicity, educational attainment and disability [13-15]. Evidence of a social gradient in physical activity by socioeconomic status (SES) is more complex [16], but one review has found that individuals in higher socioeconomic groups tend to report higher levels of physical activity than those in the lowest [17].

Governments around the world are now placing greater emphasis on developing strategies to improve population health while also reducing inequalities [18,19]. However, little is known about whether these objectives can be achieved simultaneously [20,21]. Efforts to improve average population health may be achieved without significant changes to the social distribution of health. Of greater concern is the prospect that health improvement programmes may increase overall health while inadvertently widening the gap between advantaged and disadvantaged groups, leading to so-called “intervention-generated inequalities” (IGIs) [20].

This is a particular concern for interventions seeking to tackle the social determinants of inactivity. Environmental and policy (or ‘upstream’) interventions offer a promising approach for achieving population change in physical activity [20,22,23]. However, the effects of interventions may not be evenly distributed across society and may result in most benefit accruing to the most advantaged groups, the so called “inverse care law” effect [24]. Numerous systematic reviews have examined the effectiveness of interventions to promote physical activity [25,26], and some have examined effectiveness in disadvantaged socio-economic groups, ethnic minorities or females [5,27-29]. However, there is a lack of evidence regarding the social distribution of effectiveness of environmental and policy interventions, and what evidence does exist is largely outdated [26].

Without further consideration of their distributional effects, it is impossible to evaluate whether measures to improve overall population levels of physical activity are also reducing inequalities. In this paper we report the findings of a pilot study examining how distributional effects have been reported in systematic reviews and primary studies of the effects of environmental and policy interventions. The aims of this study were to examine available evidence that may be used to answer the question; to explore appropriate techniques for synthesising evidence relating to distributional effectiveness; and to judge whether a full systematic review would be necessary and practicable.

## Methods

In order to assess the feasibility of conducting a full systematic review and to explore some of the conceptual and methodological issues likely to be encountered, a pilot review was performed to assess the availability of information on the social distribution of intervention effects, the targeting or allocation of interventions, and the baseline characteristics of participants. Pilot reviews seek to identify what research currently exists and help to inform what a full review might entail, what resources might be required and what methods might be used to synthesise the evidence [30].

### Search strategy

**Figure 1:**
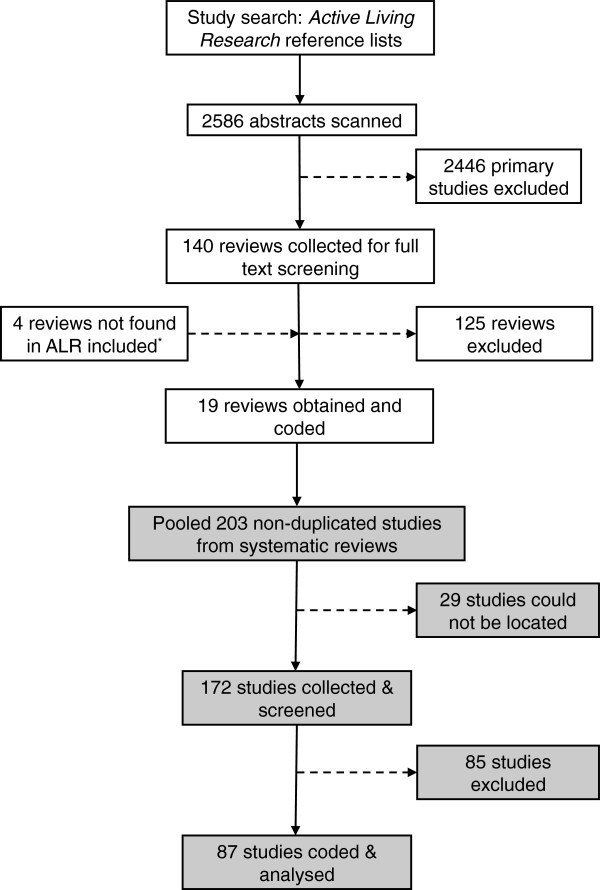
Flow diagram of selection process.

As this was a pilot study, it was necessary to balance the breadth of the search strategy and the depth of screening. Our search strategy used a secondary resource that collects and publishes citations related to active living research. This resource is maintained by researchers at the University of California, San Diego, who compile the Active Living Research (ALR) Reference lists biannually. The reference lists are compiled through systematic searches of PubMed, ISI Web of Science and various indexed and non-indexed journals [31]. We used the ALR reference lists to retrieve relevant studies using a two stage process. The first stage involved a rapid review of systematic reviews, and the second comprised a review and synthesis of a sample of primary studies included in the eligible systematic reviews (Figure 1).

### Study selection and inclusion criteria

We defined ‘environmental interventions’ as measures that change aspects of the physical environment to facilitate greater participation in physical activity. These might include changing aspects of the external physical environment such as cycle paths, bridges, road markings and walking trails, and so forth. They might also aim to change behaviour within internal physical environments such as office buildings, shopping arcades and public transport terminals, for example by encouraging stair use or installing showering or bicycle parking facilities.

‘Policy interventions’ were defined as legislative or regulatory strategies to encourage behaviour change by making unhealthy behaviours more expensive or less convenient. In this study we applied this definition broadly to include national or regional policy interventions (such as subsidies for public transport or fitness equipment, bicycle hire schemes, or congestion or parking charges) or planning initiatives. We also included organisational, workplace or school policies that provide incentives for active travel or active work, or govern the availability of supervised physical activity for children within or outside school hours.

We also included multicomponent community interventions that included either ‘environmental’ or ‘policy’ components. Although such interventions do not fit neatly within the framework of either ‘environmental’ or ‘policy’ interventions, such initiatives often incorporate blanket targeting of populations and therefore qualified under our broad definition.

### Studies were excluded if they

1. a. Were narrative or other non-systematic reviews that did not incorporate a systematic process for identifying studies for inclusion (Stage 1).

1. b. Were primary studies that were neither experimental nor quasi-experimental in design (i.e. cross-sectional studies) (Stage 2).

2 Did not examine the effects of an intervention.

3 Involved interventions that could not be characterised as containing ‘environmental’ or ‘policy’ components.

4 Did not include a measure of change in self-reported or objectively measured physical activity.

5 Were published in a language other than English.

6 If they were inaccessible at the time of data collection and analysis.

7 Were focused exclusively on disadvantaged population subgroups (e.g. low-income populations, ethnic minorities).

### Data extraction and quality assessment

In both stages of the pilot we examined whether studies reported research findings and sample characteristics by socio-demographic groupings. To do this we used the PROGRESS-Plus framework recommended by the Cochrane/Campbell Health Equity Group [32,33]. The framework includes several socio-demographic factors that may impact on health equity: place of residence, race/ethnicity, occupation, gender, religion/culture, education, socio-economic status, social capital/networks, disability, sexual orientation, and age. We examined whether subgroup intervention effects, interaction effects, adjusted associations or baseline socio-demographic characteristics were reported by any of the PROGRESS-Plus items in each of the reviews and primary studies.

Using a coding scheme adapted from Thomas et al. [34] (Table 1), primary studies were further coded on the suitability of the study design [35],the methodological execution of the study [36], and the type of outcome metric used to examine effects.

**Table 1:** Coding scheme for primary studies (adapted from Thomas et al., 2008)

**Design feature**	**Definition**	**Coding**
**a. Suitability of study design**	The height of the bar represents the level of suitability in the design of the evaluation	**Level 1 -** The study involved measurements of exposure and outcome at a single point in time
*US Task Force on Community Preventive Services*^*25*^		**Level 2 -** The study design involved single 'before' and 'after' measurements with no concurrent comparison group
		**Level 3 -** The study design included at least two 'before' measurements and at least two 'after' measurements but no concurrent comparison group
		**Level 4 -** The study design included concurrent comparison groups AND prospective measurement of exposure and outcome
**b. Methodological quality criteria**	The annotated number represents an overall score for methodological execution of the study. Studies are scored on a scale between 0–6, dependent on how many of the methodological features are achieved in each study	**Representativeness**: Were the study samples randomly recruited from the study population with a response rate of at least 60% OR were they otherwise shown to be representative of the study population?
*Effective public health practice project, Hamilton, Ontario *^*26*^		**Randomisation:** Were participants, groups or areas randomly allocated to receive the intervention or control condition?
		**Comparability:** Were the baseline characteristics of the comparison groups comparable OR if there were important differences in potential confounders were these appropriately adjusted for in the analysis? If there was no comparison group this criterion could not be met
		**Credibility of data collection instruments:** Were data collection tools shown to be credible, e.g. shown to be valid and reliable in published research, OR in a pilot study, OR taken from a published national survey, OR recognized as an acceptable measure (such as a biochemical measures of smoking)?
		**Attrition rate:** Were outcomes studied in a panel of respondents with an attrition rate of less than 30% OR were results based on a cross-sectional design with at least 200 participants included in analysis in each wave?
		**Attributability to intervention:** Is it reasonably likely that the observed effects were attributable to the intervention under investigation? This criterion could not be met if there was evidence of contamination of a control
**c. Physical activity outcome metrics**	The tone of the bars indicates what type of outcome metric was used in each study	**White** = Direct observation
		**Grey** = Self-reported measures
		**Black** = Objective measures

### Pilot synthesis

In stage 2 we used harvest plots to graphically summarise the data generated from coding the primary studies. The harvest plot method was designed to assist the synthesis of evidence on social gradients in intervention effectiveness [37]. It employs a hypothesis-testing approach in which a null hypothesis (of no social gradient) and two alternative hypotheses (of a positive or a negative social gradient) are specified and each study is coded according to which of the three competing hypotheses its results most support. A study showing that an intervention was more effective in advantaged groups is characterised as supporting a hypothesis of a ‘positive social gradient’, whereas a study showing greater effectiveness in disadvantaged groups is characterised as supporting a hypothesis of a ‘negative social gradient’. The latter would normally be considered more desirable, because it implies that a given intervention may help to reduce inequalities. Each mark on the harvest plot represents the result of a single study, weighted by the assessment of study quality.

## Results

### Stage 1: Review of reviews

The search strategy identified 19 systematic reviews that assessed the effectiveness of interventions relevant to our inclusion criteria (Additional file 1). The reviews could be grouped into five broad categories: three reviews of community interventions, three reviews of interventions to promote walking and cycling, five general reviews of physical activity interventions, three reviews of school or workplace interventions and five reviews of environmental or policy interventions.

Only five of the reviews incorporated analysis of distributional effects within their design [27,28,38-40], and only three of these were designed to synthesise the social distribution of effects. Baker et al. [38] intended to perform subgroup analysis to explore whether there was a relationship between effect and social disadvantage. Similarly, in two reviews by Ogilvie and colleagues, the authors recorded information where the social distribution of effects was reported [39,40]. However, in all three reviews the authors found that distributional effects were rarely reported and, if reported, mentioned only briefly. The two remaining reviews concentrated on assessing the effectiveness of interventions focused on disadvantaged social groups [27,28]. Neither of these were able to synthesise distributional effects because of their focus on discrete population groups, but their inclusion was important because of their potential contribution to identifying relevant primary studies.

**Figure 2:**
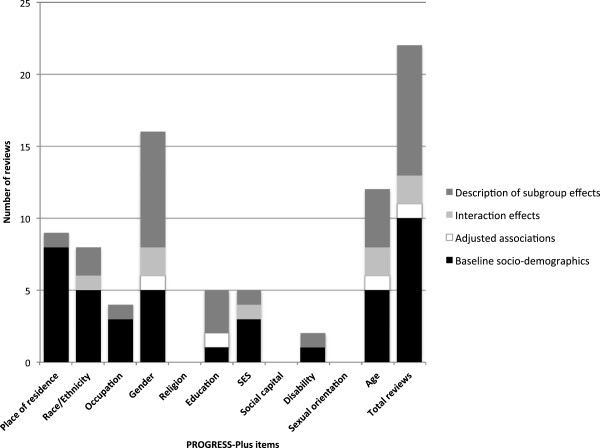
**Results of the review of reviews (n=19).** Bar counts do not sum to 19 because studies were double counted if they reported multiple types of relevant data across multiple PROGRESS-Plus items.

Although none of the included reviews synthesised distributional effects, nine provided post-hoc descriptions of intervention effects grouped by one or more of the PROGRESS-Plus items. As shown in Figure 2, the reporting of subgroup intervention effects tended to be dominated by comparisons of effectiveness between males and females. However, there was evidence that intervention effects had been compared by other PROGRESS-Plus items including age, education, ethnicity, SES, disability and place of residence. While this appears promising, such results were often reported in tabulated appendices in which the characteristics and main findings of each primary study were described only briefly without presenting effect sizes, confidence intervals or p-values. As a consequence, the consistency and comprehensiveness in reporting distributional effects tended to vary both between and within reviews.

**Figure 3:**
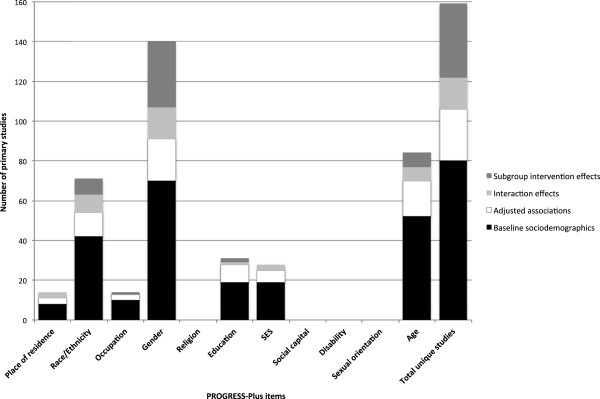
**Results of the review of primary studies (n=87).** Bar counts do not sum to 87 because studies were double counted if they reported multiple types of relevant data across multiple PROGRESS-Plus items.

Figure 3 also shows the lack of available information on adjusted associations or interaction effects contained within eligible reviews. Only two reviews reported interaction effects or adjusted associations by any of the PROGRESS-Plus items [40-42]. Foster & Hillsdon [41] occasionally reported interaction effects in their review of environmental interventions. In this case, space constraints appear to have prevented the authors from elaborating on the specific results for each study and limited any further discussion of their implications. Unlike previous reviews, Ogilvie et al. [40] presented a comprehensive summary of each primary study in an online appendix. This additional document reported specific statistical information on such adjusted associations, interaction effects and subgroup intervention effects as were reported in primary studies.

Despite the apparent absence of adjusted associations and interaction effects, Figure 2 shows that baseline information on socio-demographic characteristics of study populations was frequently reported in reviews, particularly in terms of place of residence, gender, ethnicity, occupation, age and SES. Although these characteristics tended to be reported in limited detail, the availability of these data indicates that information relevant for comparing social distributional effects is frequently collected and reported. It is unclear whether the lack of synthesis of distributional effects is due to limitations in reviews, limitations in the primary studies, or both. To investigate this further, we analysed the availability of this information in primary studies.

### Stage 2: Availability of information from primary studies

To generate a sample of primary studies, we pooled 203 studies identified from the eligible systematic reviews from stage 1. Of these studies, 29 could not be located (conference presentations, missing weblinks, etc.) but the remaining 172 studies were collected and screened. We excluded many studies that did not meet our inclusion criteria. Studies were excluded because they were not evaluations of interventions (n=42), the interventions could not be defined as ‘environmental’ or ‘policy’ (n=17) or were not targeted at the population level (n=3), the evaluations did not include a measure of physical activity (n=6), or for a variety of other reasons such as unclear descriptions or publication in a foreign language (n=17). The 87 included studies were grouped into four broad categories of intervention: changes to the built environment (n=3); multicomponent community interventions (n=14); school or workplace interventions (n=55); and behaviour prompts such as those to encourage stair use (n=15). The results of the assessment of study quality are summarised in Additional file 1.

The availability of socio-demographic information within primary studies was greater than that within the reviews (Figure 3). Although only 19 per cent (n=17) of primary studies set out to examine distributional effectiveness, over 40 per cent (n=37) reported subgroup intervention effects. These were largely confined to comparisons of effects between males and females, although some studies had considered distributional effects by ethnicity, age, education, and occupation. We also found 16 studies that reported interaction effects, and 2 studies that reported adjusted associations by one or more of the PROGRESS-Plus items. Most notably, over 90 per cent (n=80) of the primary studies reported baseline socio-demographic characteristics in all but four (religion, social capital, disability, and sexual orientation) of the PROGRESS-Plus categories. These findings reiterate the previous observation that the socio-demographic data required to investigate distributional effects are frequently collected, if not always analysed or reported.

### Evidence synthesis

**Figure 4:**
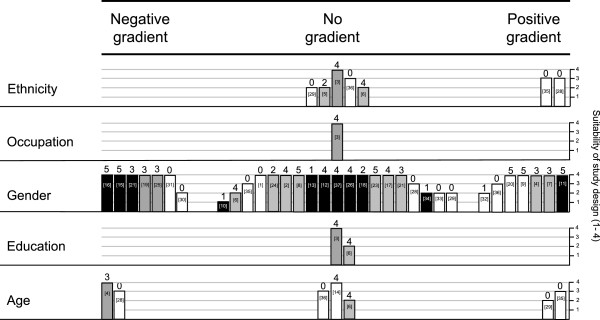
**Harvest plot of subgroup intervention effects in primary studies.** The harvest plot summarises several aspects of the primary studies. Each block represents an individual study. The positioning of each block under one of the three headings reflects which of the three competing hypotheses is most supported by the findings of each study. The height of the bars represents the *suitability of the study design* (1-4), the number above each bar represents the *study quality* (1-6), and the tone of the bars indicates the *outcome metric*, (white for direct observation, grey for self-report, and black for objective measures of physical activity). The number within each bar is that of the citation number for the study (Additional file 1).

The harvest plot in Figure 4 summarises distributional effects found in the 37 primary studies that reported subgroup intervention effects. The plot shows how each study reported information on subgroup intervention effects, for which domains of the PROGRESS-Plus framework these were reported, and how these results were distributed. The diagram also displays key information on different aspects of the study designs (Table 1).

Figure 4 shows that when studies examined differential effectiveness, they tended to do so by comparing effectiveness between males and females. On visual inspection, the findings for occupation, education and age do not appear to show any consistent evidence of a social gradient in effectiveness. For ethnicity the evidence appears to favour a positive gradient, suggesting that interventions may be more likely to benefit participants belonging to the majority ethnic group. However, this impression is based on a small number of lower-quality studies and should therefore be treated with caution. For gender, effects appear to be evenly distributed with over half of the studies (n=19) supporting the hypothesis of no social gradient in effectiveness. While several studies reported evidence suggestive of a positive social gradient, these were counterbalanced by several studies showing the opposite. This heterogeneity suggests that different interventions might affect males and females differently.

At this stage it is difficult to isolate factors that might be associated with more favourable equity impacts. Focussing on the 34 studies that compared outcomes by gender, there was no clear pattern of success (or failure) in addressing social inequalities according to type of intervention. For environmental interventions, the findings of seven studies were consistent with no differential effect by gender, while three studies found greater improvements in physical activity in males and four reported effects favouring females. For policy interventions, a similar pattern was found: 65 per cent (n=13) of studies reported no evidence for a differential effect, while 20 per cent (n=4) of studies found effects favouring males and 15 per cent (n=3) found effects favouring females. In a more extensive review it may be possible to explore the context and characteristics of interventions to further understand how and why interventions affect the social distribution of physical activity.

## Discussion

Consistent with other studies, we found that existing reviews have tended not to synthesise the distributional effectiveness of environmental and policy interventions [26]. We found numerous reviews summarising the average effectiveness of interventions. We also found several reviews that examined the effectiveness of interventions in disadvantaged groups. In broad reviews, the objective was usually restricted to assessing average population effects, whereas for focussed reviews, effects were examined only for specific population subgroups. Consequently, most reviews in either category were unable to synthesise differential effectiveness between population subgroups. While existing reviews continue to advance our understanding of the population effect of such interventions, their effect on inequalities remains largely unknown.

The lack of attention to this question is surprising considering the increased emphasis placed on tackling health inequalities over the past 15 years [43]. Some reviews have offered explanations for the absence of this evidence including a lack of exploration of effects on inequalities in reviews [26], and a lack of evidence in, or insufficient quality of, primary studies [38-40]. We found that relevant information (on subgroup intervention effects, interaction effects, adjusted associations, and baseline characteristics) was often available within primary studies. This suggests that opportunities to learn more about the effect of environmental and policy interventions on inequalities in physical activity may have been missed [33].

However, barriers to synthesising distributional effects are not restricted to the availability of data. Unlike for more traditional ‘what works’ evaluative research questions, methods for synthesising outcome data for interventions to reduce inequalities are less well developed and may even be misleading [33,44]. For example, practical limitations and research costs may prevent researchers from prospectively designing studies with sufficient power to examine distributional interaction effects across pre-specified domains [45]. As a result, evidence is often generated through post-hoc subgroup analyses, which are not based on randomised comparisons and suffer from reductions in statistical power that may inflate the likelihood of type I error and may lead to crucial misinterpretations [46]. This creates uncertainty and puts review authors in a difficult position: being encouraged to explore differential effectiveness by policymakers, yet being discouraged from performing subgroup analyses by statisticians — a contradiction that may discourage researchers from pursuing questions about inequalities [33].

In the absence of an immediate solution, we have described a short-term approach that enables cautious inferences to be made from existing research [33]. Using a harvest plot synthesis, it is possible to isolate features of environmental and policy interventions that are associated with impacts on inequalities. The ‘harvest plot’ employs a theory-driven approach to examine whether distributional effects support a particular hypothesis. The inclusion of information on the quality and rigour of primary studies enables visual communication of information on the distributional effects together with selected characteristics of each study. However, the approach we have taken is subject to some limitations, which should be addressed in future research. For example, the findings are based on a subsample of available reviews and primary studies and may therefore not be fully representative. Furthermore, these findings are tentative in that additional research is required to understand whether the inferences made about differential or null effects are statistically valid and theoretically plausible.

## Conclusions

Without greater emphasis on the distributional impact of environmental and policy interventions, it is possible that efforts to improve health may inadvertently contribute to increasing inequalities [33]. It is therefore important to understand whether interventions are effective in those who stand to benefit most from them [20,47]. In this study we found that existing systematic reviews lack sufficient information on the distributional effectiveness of environmental or policy interventions to increase participation in physical activity. We have also shown that it is both necessary and feasible to synthesise better evidence of this kind using existing data. In the longer term, however, greater emphasis should be placed on improving the design and reporting of primary research on the distributional effects of population level interventions.

## Competing interests

The authors declare that they have no competing interests.

## Authors’ contributions

Both authors contributed to the design of the study. DH executed the search strategy, screened the initial results, and appraised and extracted data from the included studies. Both authors contributed to the analysis and interpretation of the findings and drafted, revised and approved the final manuscript.

## Supplementary Material

Additional file 1 Coding Matrix for Primary Studies. Click here for file
