# Supplementary material for: Synthesising evidence for equity impacts of population-based physical activity interventions: a pilot study
Source: Int J Behav Nutr Phys Act. 2013 Jun 15;10:76. doi: 10.1186/1479-5868-10-76 (PMC3706268; doi:10.1186/1479-5868-10-76)
Supplement: Additional file 1 — Coding Matrix for Primary Studies. [file 1479-5868-10-76-S1.doc]

**Coding Matrix for Primary Studies**

|  |  |  |  | **Ethnicity** | | | **Occupation** | | | **Gender** | | | **Education** | | | **Age** | | |
| --- | --- | --- | --- | --- | --- | --- | --- | --- | --- | --- | --- | --- | --- | --- | --- | --- | --- | --- |
| **Study No.*** | **Name** | **SC** | **MQ** | **Neg** | **Nul** | **Pos** | **Neg** | **Nul** | **Pos** | **Neg** | **Nul** | **Pos** | **Neg** | **Nul** | **Pos** | **Neg** | **Nul** | **Pos** |
| 28 | Andersen et al (1998) | 3 | 0 |  |  | **☐** |  |  |  |  | **☐** |  |  |  |  | **☐** |  |  |
| 32 | Blamey et al (1995) | 2 | 1 |  |  |  |  |  |  |  |  | **☐** |  |  |  |  |  |  |
| 30 | Boutelle et al (2001) | 2 | 0 |  |  |  |  |  |  | **☐** |  |  |  |  |  |  |  |  |
| 2 | Brown 2006 | 4 | 4 |  |  |  |  |  |  |  | **◼** |  |  |  |  |  |  |  |
| 35 | Brownell et al (1980) (a) | 3 | 0 |  |  | **☐** |  |  |  |  | **☐** |  |  |  |  |  | **☐** |  |
| 36 | Brownell et al (1980) (b) | 3 | 0 |  | **☐** |  |  |  |  |  |  | **☐** |  |  |  |  | **☐** |  |
| 3 | Brownson 2004 | 4 | 4 |  | **◼** |  |  | **◼** |  |  |  |  |  | **◼** |  |  |  |  |
| 6 | Brownson et al (1996) | 2 | 4 |  | **◼** |  |  |  |  |  | **◼** |  |  | **◼** |  |  | **◼** |  |
| 24 | Cason & Logan (2006) | 4 | 2 |  |  |  |  |  |  |  | **◼** |  |  |  |  |  |  |  |
| 31 | Coleman et al (2001) | 4 | 0 |  |  |  |  |  |  | **☐** |  |  |  |  |  |  |  |  |
| 27 | Dale & Corbin (2000) | 4 | 1 |  |  |  |  |  |  |  | **◼** |  |  |  |  |  |  |  |
| 18 | Dwyer et al(1983) | 4 | 2 |  |  |  |  |  |  |  | **◼** |  |  |  |  |  |  |  |
| 4 | Eaton 1999 | 4 | 3 |  |  |  |  |  |  |  |  | **◼** |  |  |  | **◼** |  |  |
| 17 | Fardy et al (1996) | 4 | 4 |  |  |  |  |  |  |  | **◼** |  |  |  |  |  |  |  |
| 21 | Frenn & Malin (2005) | 4 | 3 |  |  |  |  |  |  |  | **◼** |  |  |  |  |  |  |  |
| 8 | Gortmaker et al 1999 | 4 | 5 |  |  |  |  |  |  |  | **◼** |  |  |  |  |  |  |  |
| 11 | Haerens et al (2006) | 4 | 5 |  |  |  |  |  |  |  |  | **◼** |  |  |  |  |  |  |
| 13 | Harris et al (1997) | 4 | 1 |  |  |  |  |  |  |  | **◼** |  |  |  |  |  |  |  |
| 12 | Kain et al (2004) | 4 | 4 |  |  |  |  |  |  |  | **◼** |  |  |  |  |  |  |  |
| 25 | Kelder et al (1993) | 4 | 3 |  |  |  |  |  |  | **◼** |  |  |  |  |  |  |  |  |
| 29 | Kerr et al (2001) | 2 | 0 |  | **☐** |  |  |  |  |  | **☐** |  |  |  |  |  |  | **☐** |
| 37 | Killen et al (1988) | 4 | 4 |  |  |  |  |  |  |  | **◼** |  |  |  |  |  |  |  |
| 26 | Killen et al (1989) | 4 | 4 |  |  |  |  |  |  |  | **◼** |  |  |  |  |  |  |  |
| 7 | Lupton et al (2003) | 4 | 3 |  |  |  |  |  |  |  |  | **◼** |  |  |  |  |  |  |
| 20 | McKenzie et al (2004) | 4 | 5 |  |  |  |  |  |  |  |  | **☐** |  |  |  |  |  |  |
| * Study numbers correspond to Harvest Plot, SC = Suitability of study design (1-4), MQ = Methodological quality (1-6) | | | | | | | | | | | | | | | | | | |

Table 1A Continued.

|  | | | | | | | | | | | | | | | | | | |
| --- | --- | --- | --- | --- | --- | --- | --- | --- | --- | --- | --- | --- | --- | --- | --- | --- | --- | --- |
|  |  |  |  | **Ethnicity** | | | **Occupation** | | | **Gender** | | | **Education** | | | **Age** | | |
| **Study No.** | **Name** | **SC** | **MQ** | **Neg** | **Nul** | **Pos** | **Neg** | **Nul** | **Pos** | **Neg** | **Nul** | **Pos** | **Neg** | **Nul** | **Pos** | **Neg** | **Nul** | **Pos** |
| 5 | Merom et al (2003) | 2 | 2 |  | **◼** |  |  |  |  |  |  |  |  |  |  |  |  |  |
| 1 | Painter (1996) | 4 | 0 |  |  |  |  |  |  |  | **☐** |  |  |  |  |  |  |  |
| 10 | Pangrazi et al (2003) | 1 | 1 |  |  |  |  |  |  |  | **◼** |  |  |  |  |  |  |  |
| 19 | Perry et al (1987) | 4 | 3 |  |  |  |  |  |  | **◼** |  |  |  |  |  |  |  |  |
| 15 | Sallis et al (1997) | 4 | 5 |  |  |  |  |  |  | **◼** |  |  |  |  |  |  |  |  |
| 9 | Sallis et al (2003) | 4 | 5 |  |  |  |  |  |  |  |  | **☐** |  |  |  |  |  |  |
| 34 | Shepherd (2001) | 2 | 1 |  |  |  |  |  |  |  | **◼** |  |  |  |  |  |  |  |
| 23 | Simon et al (2006) | 4 | 5 |  |  |  |  |  |  |  | **◼** |  |  |  |  |  |  |  |
| 14 | Simons-Morton et al (1991) | 4 | 4 |  |  |  |  |  |  |  |  |  |  |  |  |  | **☐** |  |
| 33 | Titze et al (2001) | 2 | 0 |  |  |  |  |  |  |  | **☐** |  |  |  |  |  |  |  |
| 16 | Vandongen et al (1995) | 4 | 5 |  |  |  |  |  |  | **◼** |  |  |  |  |  |  |  |  |
| 22 | Vestraete et al (2006) | 4 | 3 |  |  |  |  |  |  | **◼** |  |  |  |  |  |  |  |  |
| SC = Suitability category (1-4), MQ = Methodological quality (1-6) | | | | | | | | | | | | | | | | | | |

**References for Review Studies (RW^#^):**

^RW^1. Baker PRA, Francis DP, Soares J, Weightman AL, Foster C. Community wide interventions for increasing physical activity. Cochrane Database of Systematic Reviews. 2011(4):436-7.

^RW^2. Yang L, Sahlqvist S, McMinn A, Griffin SJ, Ogilvie D. Interventions to promote cycling: systematic review. BMJ: British Medical Journal. 2010;341.

^RW^3. Timperio A, Salmon J, Ball K. Evidence-based strategies to promote physical activity among children, adolescents and young adults: review and update. Journal of Science and Medicine in Sport. 2004;7(1):20-9.

^RW^4. Zenzen W, Kridli S. Integrative review of school-based childhood obesity prevention programs. Journal of Pediatric health care. 2009;23(4):242-58.

^RW^5. Hoehner CM, Soares J, Parra Perez D, Ribeiro IC, Joshu CE, Pratt M, et al. Physical Activity Interventions in Latin America:: A Systematic Review. American Journal of Preventive Medicine. 2008;34(3):224-33.

^RW^6. Heath GW, Brownson RC, Kruger J, Miles R, Powell KE, Ramsey LT. The effectiveness of urban design and land use and transport policies and practices to increase physical activity: a systematic review. Journal of Physical Activity & Health. 2006;3:55.

^RW^7. de-Bruijn, G.-J., Kremers, S., Wendel-Vos, W., van Lenthe, F., Brug, J. (2005). Environmental interventions on physical activity in youth. In J. Brug & F. van Lenthe (Eds.), *Environmental determinants and intervnetions for physical activity, nutrition and smoking: A review*. Maastricht: Universiteit Maastrict.

^RW^8. Kremers, S., de Buijn, G.-J., Wendel-Vos, W., van Lenthe, F., Brug, J. (2005). Environmental interventions on physical activity in adults. In J. Brug & F. van Lenthe (Eds.), *Environmental determinants and interventions for physical activity, nutrition and smoking: A review*. Maastricht: Universiteit Maastrict.

^RW^9. Engbers LH, Van Poppel MNM, Chin A Paw MJM, van Mechelen W. Worksite health promotion programs with environmental changes: a systematic review. American Journal of Preventive Medicine. 2005;29(1):61-70.

^RW^10. Ogilvie D, Egan M, Hamilton V, Petticrew M. Promoting walking and cycling as an alternative to using cars: systematic review. BMJ. 2004;329(7469):763.

^RW^11. Foster C, Hillsdon M. Changing the environment to promote health-enhancing physical activity. Journal of Sports Sciences. 2004;22(8):755-69.

^RW^12. Kahn EB, Ramsey LT, Brownson RC, Heath GW, Howze EH, Powell KE, et al. The effectiveness of interventions to increase physical activity:: A systematic review1 and 2. American Journal of Preventive Medicine. 2002;22(4):73-107.

^RW^13. Yancey AK, Kumanyika SK, Ponce NA, McCarthy WJ, Fielding JE, Leslie JP, et al. Population-based Interventions Engaging Communities of Color in Healthy Eating and Active Living: A Review. Preventing chronic disease: public health research, practice, and policy. 2004;1(1):1-18.

^RW^14. Banks-Wallace J, Conn V. Interventions to promote physical activity among African American women. Public Health Nursing. 2002;19(5):321-35.

^RW^15. Fogelholm M, Lahti-Koski M. Community health-promotion interventions with physical activity: does this approach prevent obesity? Food & Nutrition Research. 2002;46(4):173-7.

^RW^16. Kropski JA, Keckley PH, Jensen GL. School-based obesity prevention programs: an evidence-based review. Obesity. 2008;16(5):1009-18.

^RW^17. Pucher J, Dill J, Handy S. Infrastructure, programs, and policies to increase bicycling: An international review. Preventive Medicine. 2010;50:106-25.

^RW^18. Salmon J, Booth ML, Phongsavan P, Murphy N, Timperio A. Promoting physical activity participation among children and adolescents. Epidemiologic reviews. 2007;29(1):144-59.

^RW^19. Ogilvie D, Foster CE, Rothnie H, Cavill N, Hamilton V, Fitzsimons CF, et al. Interventions to promote walking: systematic review. BMJ. 2007;334:1204.

| **Table 2A: Results for review of reviews (n=19)** | | | | | |
| --- | --- | --- | --- | --- | --- |
|  | **Baseline socio-demographics** | **Adjusted associations** | **Interaction effects** | **Description of subgroup intervention effects** | **Total reviews** |
| **Place of residence** | [4,6,7,8,9,10,11,15,19] |  |  | [8] | 9 |
| **Race/Ethnicity** | [6,7,8,9,18] |  | [19] | [8,11] | 7 |
| **Occupation** | [9,10,11] |  |  | [10] | 3 |
| **Gender** | [6,7,11,18,19] | [11] | [11,19] | [1,7,8,10,11,16,18,19] | 9 |
| **Religion** |  |  |  |  | 0 |
| **Education** | [6] | [11] |  | [1,8,11,19] | 5 |
| **SES** | [4,6,18] |  | [19] | [19] | 4 |
| **Social capital** |  |  |  |  | 0 |
| **Disability** | [6] |  |  | [10] | 2 |
| **Sexual orientation** |  |  |  |  | 0 |
| **Age** | [4,6,7,18,19] | [11] | [11,19] | [1,10,11,18] | 9 |
| **Total reviews** | 10 | 1 | 2 | 9 |  |

**References for Primary Studies (P^#^):**

^P^1. Brown WJ, Mummery K, Eakin E, Schofield G. 10,000 Steps Rockhampton: evaluation of a whole community approach to improving population levels of physical activity. Journal of Physical Activity & Health. 2006;3(1):1.

^P^2. Simon C, Schweitzer B, Oujaa M, Wagner A, Arveiler D, Triby E, et al. Successful overweight prevention in adolescents by increasing physical activity: a 4-year randomized controlled intervention. International Journal of Obesity. 2008;32(10):1489-98.

^P^3. Brownson RC, Baker EA, Boyd RL, Caito NM, Duggan K, Housemann RA, et al. A community-based approach to promoting walking in rural areas. American Journal of Preventive Medicine. 2004;27(1):28-34.

^P^4. De Cocker KA, De Bourdeaudhuij IM, Brown WJ, Cardon GM. Effects of "10,000 steps Ghent": a whole-community intervention. American Journal of Preventive Medicine. 2007;33(6):455-63.

^P^5. Eaton C, Lapane K, Garber C, Gans K, Lasater T, Carleton R. Effects of a community-based intervention on physical activity: the Pawtucket Heart Health Program. American journal of public health. 1999;89(11):1741-4.

^P^6. Jenum AK, Anderssen SA, Birkeland KI, Holme I, Graff-Iversen S, Lorentzen C, et al. Promoting physical activity in a low-income multiethnic district: effects of a community intervention study to reduce risk factors for type 2 diabetes and cardiovascular disease. Diabetes Care. 2006;29(7):1605-12.

^P^7. NSW Health. Walk it, active local parks: The effect of park modifications and promotion on physical activity participation Sydney: New South Wales Department of Health; 2002.

^P^8. O'Loughlin JL, Paradis G, Gray-Donald K, Renaud L. The impact of a community-based heart disease prevention program in a low-income, inner-city neighborhood. American journal of public health. 1999;89(12):1819-26.

^P^9. Gortmaker Sl, Peterson K, Wiecha J, Sobol AM, Dixit S, Kay Fox M, et al. Reducing obesity via a school-based interdisciplinary intervention among youth: Planet health. Archives of Pediatrics & Adolescent Medicine. 1999;153(4):409-18.

^P^10. Rowland D, DiGuiseppi C, Gross M, Afolabi E, Roberts I. Randomised controlled trial of site specific advice on school travel patterns. Archives of disease in childhood. 2003;88(1):8-11.

^P^11. Manios Y, Moschandreas J, Hatzis C, Kafatos A. Health and nutrition education in primary schools of Crete: changes in chronic disease risk factors following a 6-year intervention programme. British Journal of Nutrition. 2002;88(3):315-24.

^P^12. Harrell JS, McMurray RG, Gansky SA, Bangdiwala SI, Bradley CB. A public health vs a risk-based intervention to improve cardiovascular health in elementary school children: the Cardiovascular Health in Children Study. American journal of public health. 1999;89(10):1529-35.

^P^13. Sallis JF, McKenzie TL, Conway TL, Elder JP, Prochaska JJ, Brown M, et al. Environmental interventions for eating and physical activity: A randomized controlled trial in middle schools. American Journal of Preventive Medicine. 2003;24(3):209-17.

^P^14. Beurden Ev, Barnett LM, Zask A, Dietrich UC, Brooks LO, Beard J. Can we skill and activate children through primary school physical education lessons? "Move it groove it" - a collaborative health promotion intervention. Preventive Medicine. 2003;36(4):493-501.

^P^15. Pangrazi RP, Beighle A, Vehige T, Vack C. Impact of Promoting Lifestyle Activity for Youth (PLAY) on children's physical activity. Journal of School Health. 2003;73(8):317-21.

^P^16. Sahota P, Rudolf MCJ, Dixey R, Hill AJ, Barth JH, Cade J. Randomised controlled trial of primary school based intervention to reduce risk factors for obesity. BMJ. 2001;323(7320):1029.

^P^17. Warren JM, Henry CJK, Lightowler HJ, Bradshaw SM, Perwaiz S. Evaluation of a pilot school programme aimed at the prevention of obesity in children. Health Promotion International. 2003 December 1, 2003;18(4):287-96.

^P^18. Caballero B, Clay T, Davis SM, Ethelbah B, Rock BH, Lohman T, et al. Pathways: a school-based, randomized controlled trial for the prevention of obesity in American Indian schoolchildren. The American journal of clinical nutrition. 2003;78(5):1030-8.

^P^19. Coleman KJ, Tiller CL, Sanchez J, Heath EM, Sy O, Milliken G, et al. Prevention of the epidemic increase in child risk of overweight in low-income schools: the El Paso coordinated approach to child health. Archives of Pediatrics and Adolescent Medicine. 2005;159(3):217.

^P^20. Frenn M, Malin S, Bansal N, Delgado M, Greer Y, Havice M, et al. Addressing Health Disparities in Middle School Students' Nutrition and Exercise. Journal of Community Health Nursing. 2003;20(1):1-14.

^P^21. Haerens L, Deforche B, Maes L, Cardon G, Stevens V, De Bourdeaudhuij I. Evaluation of a 2-year physical activity and healthy eating intervention in middle school children. Health education research. 2006;21(6):911-21.

^P^22. Kain J, Uauy R, Albala FV, Cerda R, Leyton B. School-based obesity prevention in Chilean primary school children: methodology and evaluation of a controlled study. International Journal of Obesity. 2004;28(4):483-93.

^P^23. Painter K. The influence of street lighting improvements on crime, fear and pedestrian street use, after dark. Landscape and Urban Planning. 1996;35(2-3):193-201.

^P^24. Harris KJ, Paine-Andrews A, Richter KP, Lewis RK, Johnston JA, James V, et al. Reducing Elementary School Children's Risks for Chronic Diseases through School Lunch Modifications, Nutrition Education, and Physical Activity Interventions. Journal of Nutrition Education. 1997;29(4):196-202.

^P^25. Trevino RP, Pugh JA, Hernandez AE, Menchaca VD, Ramirez RR, Mendoza M. Bienestar: A Diabetes Risk-Factor Prevention Program. Journal of School Health. 1998;68(2):62-7.

^P^26. Hopper CA, Munoz KD, Gruber MB, MacConnie S, Schonfeldt B, Shunk T. A School-Based Cardiovascular Exercise and Nutrition Program With Parent Participation: An Evaluation Study. Children's Health Care. 1996 1996/07/01;25(3):221-35.

^P^27. Simons-Morton BG, Parcel GS, Baranowski T, Forthofer R, O'Hara NM. Promoting physical activity and a healthful diet among children: results of a school-based intervention study. American journal of public health. 1991;81(8):986-91.

^P^28. Sallis JF, McKenzie TL, Alcaraz JE, Kolody B, Faucette N, Hovell MF. The effects of a 2-year physical education program (SPARK) on physical activity and fitness in elementary school students. Sports, Play and Active Recreation for Kids. American journal of public health. 1997;87(8):1328-34.

^P^29. Vandongen R, Jenner DA, Thompson C, Taggart AC, Spickett EE, Burke V, et al. A Controlled Evaluation of a Fitness and Nutrition Intervention Program on Cardiovascular Health in 10-Year-Old to 12-Year-Old Children. Preventive Medicine. 1995;24(1):9-22.

^P^30. Manios Y, Moschandreas J, Hatzis C, Kafatos A. Evaluation of a Health and Nutrition Education Program in Primary School Children of Crete over a Three-Year Period. Preventive Medicine. 1999;28(2):149-59.

^P^31. Nader PR, Sellers DE, Johnson CC, Perry CL, Stone EJ, Cook KC, et al. The Effect of Adult Participation in a School-Based Family Intervention to Improve Children's Diet and Physical Activity: The Child and Adolescent Trial for Cardiovascular Health. Preventive Medicine. 1996;25(4):455-64.

^P^32. Fardy PS, White REC, Haltiwanger-Schmitz K, Magel JR, McDermott KJ, Clark LT, et al. Coronary disease risk factor reduction and behavior modification in minority adolescents: The PATH program. Journal of Adolescent Health. 1996;18(4):247-53.

^P^33. Ewart CK, Young DR, Hagberg JM. Effects of school-based aerobic exercise on blood pressure in adolescent girls at risk for hypertension. American journal of public health. 1998;88(6):949-51.

^P^34. Jamner MS, Spruijt-Metz D, Bassin S, Cooper DM. A controlled evaluation of a school-based intervention to promote physical activity among sedentary adolescent females: project FAB. Journal of Adolescent Health. 2004;34(4):279-89.

^P^35. Andersen RE, Franckowiak SC, Snyder J, Bartlett SJ, Fontaine KR. Can Inexpensive Signs Encourage the Use of Stairs? Results from a Community Intervention. Annals of Internal Medicine. 1998;129(5):363-9.

^P^36. Kerr J, Eves F, Carroll D. Posters can prompt less active people to use the stairs. Journal of Epidemiology and Community Health. 2000;54(12):942-3.

^P^37. Kerr J, Eves F, Carroll D. Six-month observational study of prompted stair climbing. Preventive Medicine. 2001;33(5):422-7.

^P^38. Kerr J, Eves FF, Carroll D. The influence of poster prompts on stair use: The effects of setting, poster size and content. British Journal of Health Psychology. 2001;6(4):397-405.

^P^39. Linenger JM, Chesson I, Charles V, Nice D. Physical fitness gains following simple environmental change. In: Center NHR, editor. San Diego, California: Naval Medical Research and Development Command; 1990.

^P^40. Titze S, Martin BW, Seiler R, Marti B. A worksite intervention module encouraging the use of stairs: results and evaluation issues. Sozial-und Präventivmedizin/Social and Preventive Medicine. 2001;46(1):13-9.

^P^41. Boutelle KN, Jeffery RW, Murray DM, Schmitz MKH. Using Signs, Artwork, and Music to Promote Stair Use in a Public Building. American journal of public health. 2001;91(12):2004-6.

^P^42. Yancey AK, McCarthy WJ, Taylor WC, Merlo A, Gewa C, Weber MD, et al. The Los Angeles Lift Off: a sociocultural environmental change intervention to integrate physical activity into the workplace. Preventive Medicine. 2004;38(6):848-56.

^P^43. Coleman KJ, Gonzalez EC. Promoting Stair Use in a US-Mexico Border Community. American journal of public health. 2001;91(12):2007-9.

^P^44. Kerr J, Eves F, Carroll D. Encouraging stair use: stair-riser banners are better than posters. American journal of public health. 2001;91(8):1192.

^P^45. Blamey A, Mutrie N, Tom A. Health promotion by encouraged use of stairs. BMJ. 1995;311(7000):289-90.

^P^46. Merom D, Bauman A, Vita P, Close G. An environmental intervention to promote walking and cycling-the impact of a newly constructed Rail Trail in Western Sydney. Preventive Medicine. 2003;36(2):235-42.

^P^47. Eddy, J.M., Eynon, D., Nagy, S., & Paradossi, P.J. (1990). Impact of a physical fitness program in a blue-collar workforce. Health Values, 14, 6, 14-2.

^P^48. Dwyer T, Coonan WE, Leitch DR, Hetzel BS, Baghurst R. An investigation of the effects of daily physical activity on the health of primary school students in South Australia. International Journal of Epidemiology. 1983;12(3):308-13.

^P^49. McKenzie TL, Nader PR, Strikmiller PK, Yang M, Stone EJ, Perry CL, et al. School Physical Education: Effect of the Child and Adolescent Trial for Cardiovascular Health. Preventive Medicine. 1996;25(4):423-31.

^P^50. Brownson RC, Smith CA, Pratt M, Mack NE, Jackson-Thompson J, Dean CG, et al. Preventing cardiovascular disease through community-based risk reduction: the Bootheel Heart Health Project. American journal of public health. 1996;86(2):206-13.

^P^51. Nader PR, Stone EJ, Lytle LA, Perry CL, Osganian SK, Kelder S, et al. Three-year maintenance of improved diet and physical activity: The catch cohort. Archives of Pediatrics & Adolescent Medicine. 1999;153(7):695-704.

^P^52. Bush PJ, Zuckerman AE, Theiss PK, Taggert VS, Horowitz C, Sheridan MJ, et al. Cardiovascular risk factor prevention in black schoolchildren: Two-tear results of the "Know your body" program. American Journal of Epidemiology. 1989 March 1, 1989;129(3):466-82.

^P^53. Perry CL, Klepp K-I, Halper A, Dudovitz B, Golden D, Griffin G, et al. Promoting healthy eating and physical activity patterns among adolescents: a pilot study of 'Slice of Life'. Health education research. 1987 June 1, 1987;2(2):93-103.

^P^54. Robinson TN. Reducing children's television viewing to prevent obesity. JAMA: the journal of the American Medical Association. 1999;282(16):1561-7.

^P^55. Bayne-Smith M, Fardy PS, Azzollini A, Magel J, Schmitz KH, Agin D. Improvements in Heart Health Behaviors and Reduction in Coronary Artery Disease Risk Factors in Urban Teenaged Girls Through a School-Based Intervention: The PATH Program. American journal of public health. 2004;94(9):1538-43.

^P^56. Harrell JS, McMurray RG, Bangdiwala SI, Frauman AC, Gansky SA, Bradley CB. Effects of a school-based intervention to reduce cardiovascular disease risk factors in elementary-school children: the Cardiovascular Health in Children (CHIC) study. The Journal of pediatrics. 1996;128(6):797-805.

^P^57. McKenzie TL, Sallis JF, Prochaska JJ, Conway TL, Marshall SJ, Rosengard P. Evaluation of a two-year middle-school physical education intervention: M-SPAN. Medicine & Science in Sports & Exercise. 2004;36(8):1382.

^P^58. Frenn M, Malin S, Brown RL, Greer Y, Fox J, Greer J, et al. Changing the tide: an Internet/video exercise and low-fat diet intervention with middle-school students. Applied Nursing Research. 2005;18(1):13-21.

^P^59. Stratton G, Mullan E. The effect of multicolor playground markings on children's physical activity level during recess. Preventive Medicine. 2005;41(5-6):828-33.

^P^60. Verstraete SJM, Cardon GM, De Clercq DLR, De Bourdeaudhuij IMM. Increasing children's physical activity levels during recess periods in elementary schools: the effects of providing game equipment. The European Journal of Public Health. 2006 August 2006;16(4):415-9.

^P^61. Simon C, Wagner A, Platat C, Arveiler D, Schweitzer B, Schlienger JL, et al. ICAPS: a multilevel program to improve physical activity in adolescents. Diabetes & Metabolism. 2006;32(1):41-9.

^P^62. Boarnet MG, Anderson CL, Day K, McMillan T, Alfonzo M. Evaluation of the California Safe Routes to School legislation: urban form changes and children's active transportation to school. American Journal of Preventive Medicine. 2005;28(2):134-40.

^P^63. Marcus AC, Channing Wheeler R, Cullen JW, Crane LA. Quasi-experimental evaluation of the Los Angeles know your body program: Knowledge, beliefs, and self-reported behaviors. Preventive Medicine. 1987;16(6):803-15.

^P^64. Pate RR, Saunders RP, Ward DS, Felton G, Trost SG, Dowda M. Evaluation of a community-based intervention to promote physical activity in youth: lessons from Active Winners. American Journal of Health Promotion. 2003;17(3):171-82.

^P^65. Cason KL, Logan BN. Educational Intervention Improves 4th-Grade Schoolchildren's Nutrition and Physical Activity Knowledge and Behaviors. Topics in Clinical Nutrition. 2006;21(3):234-40.

^P^66. Luepker RV, Perry CL, McKinlay SM, Nader PR, Parcel GS, Stone EJ, et al. Outcomes of a field trial to improve children's dietary patterns and physical activity. JAMA: the journal of the American Medical Association. 1996;275(10):768-76.

^P^67. Donnelly J, Jacobsen D, Whatley J, Hill J, Swift L, Cherrington A, et al. Nutrition and physical activity program to attenuate obesity and promote physical and metabolic fitness in elementary school children. Obesity Research. 1996;4(3):229.

^P^68. Kelder SH, Perry CL, Klepp K-I. Community-Wide Youth Exercise Promotion: Long-Term Outcomes of the Minnesota Heart Health Program and the Class of 1989 Study. Journal of School Health. 1993;63(5):218-23.

^P^69. Heirich MA, Foote A, Erfurt JC, Konopka B. Work-Site Physical Fitness Programs: Comparing the Impact of Different Program Designs on Cardiovascular Risks. Journal of Occupational and Environmental Medicine. 1993;35(5):510-7.

^P^70. Vuori IM, Oja P, Paronen O. Physically active commuting to work-testing its potential for exercise promotion. Medicine & Science in Sports & Exercise. 1994;26(7):844-50.

^P^71. Bertera RL. Behavioral risk factor and illness day changes with workplace health promotion: two-year results. American Journal of Health Promotion. 1993;7(5):365-73.

^P^72. Henritze J, Brammell HL, McGloin J. LIFECHECK: a successful, low touch, low tech, in-plant, cardiovascular disease risk identification and modification program. American Journal of Health Promotion. 1992;7(2):129-36.

^P^73. Lupton BS, Fønnebø V, Søgaard AJ. The Finnmark Intervention Study: is it possible to change CVD risk factors by community-based intervention in an Arctic village in crisis? Scandinavian Journal of Public Health. 2003 May 1, 2003;31(3):178-86.

^P^74. Russell WD, Dzewaltowski DA, Ryan GJ. The effectiveness of a point-of-decision prompt in deterring sedentary behavior. American Journal of Health Promotion. 1999;13(5):257-9.

^P^75. Russell WD, Hutchinson J. Comparison of health promotion and deterrent prompts in increasing use of stairs over escalators. Perceptual and motor skills. 2000;91(1):55-61.

^P^76. Kronenfeld JJ, Jackson K, Blair SN, Davis K, Dell Gimarc J, Salisbury Z, et al. Evaluating Health Promotion: A Longitudinal Quasi-Experimental Design. Health Education & Behavior. 1987 June 1, 1987;14(2):123-39.

^P^77. Parcel GS, Simons-Morton B, O'Hara NM, Baranowski T, Wilson B. School Promotion of Healthful Diet and Physical Activity: Impact on Learning Outcomes and Self-Reported Behavior. Health Education & Behavior. 1989 June 1, 1989;16(2):181-99.

^P^78. Fairclough S, Stratton G. Effects of a physical education intervention to improve student activity levels. Physical Education and Sport Pedagogy. 2006;11(1):29-44.

^P^79. Goldfine BD, Nahas MV. Incorporating Health-Fitness Concepts in Secondary Physical Education Curricula. Journal of School Health. 1993;63(3):142-6.

^P^80. Killen JD, Telch MJ, Robinson TN, Maccoby N, Taylor C, Farquhar JW. Cardiovascular disease risk reduction for tenth graders: A multiple-factor school-based approach. JAMA: the journal of the American Medical Association. 1988;260(12):1728-33.

^P^81. Agron P, Takada E, Purcell A. California Project LEAN's Food on the Run program: an evaluation of a high school-based student advocacy nutrition and physical activity program. Journal of the American Dietetic Association. 2002;102(3 Suppl):S103.

^P^82. Larsen P, Simons N. Evaluating a federal health and fitness program: indicators of improving health. AAOHN journal: official journal of the American Association of Occupational Health Nurses. 1993;41(3):143.

^P^83. Shepherd RJ. Twelve years experience of a fitness program for the salaried employees of a Toronto life assurance company. American Journal of Health Promotion. 1992;6(4):292-301.

^P^84. Dale D, Corbin CB. Physical activity participation of high school graduates following exposure to conceptual or traditional physical education. Research Quarterly for Exercise and Sport. 2000;71(1):61.

^P^85. Brownell KD, Stunkard AJ, Albaum JM. Evaluation and modification of exercise patterns in the natural environment. The American Journal of Psychiatry; The American Journal of Psychiatry. 1980.

^P^86. Brownell KD, Stunkard AJ, Albaum JM. Evaluation and modification of exercise patterns in the natural environment. The American Journal of Psychiatry; The American Journal of Psychiatry. 1980.

^P^87. Blair SN, Piserchia PV, Wilbur CS, Crowder JH. A public health intervention model for work-site health promotion: Impact on exercise and physical fitness in a health promotion plan after 24 months. JAMA: the journal of the American Medical Association. 1986;255(7):921-6.

^P^88. Killen JD, Robinson TN, Telch MJ, Saylor KE. The Stanford Adolescent Heart Health Program. Health Education Quarterly; Health Education Quarterly. 1989.

| **Table 3A: Results from primary studies** | | | | | |
| --- | --- | --- | --- | --- | --- |
|  | **Baseline socio-demographics** | **Adjusted associations** | **Interaction effects** | **Subgroup intervention effects** | **Total studies** |
| **Place of residence** | [3,10,12,19,31,49,51,61] | [10,49,51] | [18,31,49] |  | 9 |
| **Race/Ethnicity** | [3,6,8,9,10,12,19,20,27,28,31,32,33,34,35,37,39,44,46,48,49,50,51,54,55,56,58,62,63,64,65,66,67,71,72,73,77,80,81,85,86,88] | [3,8,9,10,12,39,44,49,50,51,56,63] | [7,12,20,31,33,49,51,85,86] | [3,35,37,46,50,58,85,86] | 43 |
| **Occupation** | [1,3,4,6,8,10,46,47,61,87] | [1,10,61] |  | [3] | 10 |
| **Gender** | [1,2,3,4,6,8,9,10,11,14,15,16,17,19,20,21,22,25,26,28,29,30,31,32,33,34,35,36,37,38,39,40,41,42,43,44,45,46,47,48,49,50,51,52,53,54,56,57,58,59,60,61,63,64,65,68,71,72,73,74,75,79,80,81,82,83,84,85,86,88] | [1,3,8,9,10,11,12,14,16,30,39,44,49,50,51,52,54,60,63,68,87] | [2,7,12,18,20,29,31,38,49,51,59,64,74,85,86,87] | [1,5,9,13,15,21,22,23,24,28,29,32,35,37,40,41,43,45,48,50,53,57,60,61,65,68,73,80,83,84,85,86,88] | 79 |
| **Religion** |  |  |  |  | 0 |
| **Education** | [1,3,4,6,8,10,11,12,20,40,46,50,54,56,71,73,75,76,87] | [1,3,4,8,10,11,12,50,56] | [20] | [3,50] | 19 |
| **SES** | [2,10,19,20,21,25,30,42,52,54,56,58,61,64,67,72,80,87,88] | [10,21,30,52,56,61] | [2,7,20] |  | 20 |
| **Social capital** |  |  |  |  | 0 |
| **Disability** |  |  |  |  | 0 |
| **Sexual orientation** |  |  |  |  | 0 |
| **Age** | [1,2,3,4,6,8,9,12,15,16,17,20,21,22,25,26,27,35,36,37,39,40,42,44,50,52,54,55,58,59,60,61,63,65,66,68,71,72,73,74,75,76,79,80,81,82,83,84,85,86,87,88] | [1,3,4,8,9,14,16,21,28,39,44,50,52,54,61,63,68,87] | [2,20,59,75,85,86,87] | [5,27,35,37,50,85,86] | 55 |
| **Total studies** | 80 | 26 | 16 | 37 |  |
